# Acknowledgments

**DOI:** 10.1111/1759-7714.12401

**Published:** 2016-11-02

The Editors wish to thank the following reviewers of manuscripts for their contribution in 2016.

Zeinab Abdel Hafeez

Sherif AbdelWahab

Krzysztof Adamowicz

Huber Adrian

Abbas Agaimy

Rupali Aggarwal

M J Ahn

Myung‐Ju Ahn

Tevfik Akcam

Huseyin Akdeniz

Okan Akhan

Aykut Aktas

Marcus Albert

Ihsan Alloubi

Levent Alpay

Tong‐Tong An

José L.F. Antunes

Takatoshi Aoki

Hiromasa Arai

Takuya Araki

Laura Ardelean

Douglas Arenberg

Keisuke Asakura

Kazuhiro Asami

Fouad Atoini

Ertan Aydin

JS Azagra

Koichi Azuma

George D. Bablekos

Emin Bagriacik

Irina Baimatova

Alessandro Baisi

Jean‐Marc Baste

Meltem Baykara

Antonio Paolo Beltrami

Kfir Ben‐David

Ioana Berindan‐Neagoe

Luca Bertolaccini

Shreekant Bharti

Andrea Billè

Michele Bisceglia

Mariano Bizzarri

Markus Blaukovitsch

Johannes Bodner

Laura Bonanno

R. Borie

Zouhour Bourhaleb

Pierre‐Yves Brichon

Tamara Brown

Ayesha Bryant

Ufuk Cagirici

Li Cai

Qian Cai

Andreea Cătană

Chee‐Yin Chai

Ying Chai

Oscar S.H. Chan

Stephen L Chan

Dong Chang

Gee‐Chen Chang

Joe Y Chang

Jung Hyun Chang

Yeun‐Chung Chang

Yoon Soo Chang

Yin Kai Chao

Guowei Che

Hyun Keun Chee

Chang Chen

Chun Chen

Long‐Bang Chen

Ying‐Yi Chen

Bharadwaj Cheruvu

Alexander Chi

Kwan‐Hwa Chi

Chih‐Feng Chian

Chu‐Chun Chien

Ying‐Chun Chien

Jong Ho Cho

Moon‐June Cho

Hayoung Choi

Hong‐Shik Choi

Kuan‐Chih Chow

Jin‐Haeng Chung

Soo Kyo Chung

Ugo Cioffi

Necati Citak

Maria Teresa Congedo

J.‐M. Cosset

Sébastien Couraud

Matthew Crabtree

Gilles Créhange

Marika Crohns

Saurabh Dahiya

Huaping Dai

Megan E. Daly

Sebastian Dango

Magdolna Dank

Anshuman Darbari

Laleng M. Darlong

Satya Das

Fabio Davoli

Sophie J. R. de Danschutter

Ramon Andrade de Mello

Serkan degirmencioglu

Giorgio Della Rocca

Nalan Demir

Ilhan Demirci

Monika Deshpande

Raj Dhupar

Quentin Diot

Brett Doble

Talha Dogruyol

Ayşe Durnalı

Anton Dzian

Takahiro Ebata

Konstantinos Economopoulos

Ibrahim Elghissassi

Katsura Emoto

Bulent Erdogan

Takahisa Eriguchi

Matthew Evison

Serdar Evman

Elizabeth Fabre

G Falco

Zhen Fan

Hsin‐Yuan Fang

Wentao Fang

Valter Nilton Felix

Po‐Hao Feng

Jose Fernando Val‐Bernal

Piero Ferolla

Diego Ferone

David J. Finley

Frédéric Fiteni

Michael Fleischhacker

A Font

Christophoros Foroulis

Richard K. Freeman

Jianhua Fu

Sidney W. Fu

Minoru Fukuda

Hiroshi Fushiki

Andrea Casadei Gardini

Ming‐Jian Ge

V. Georgoulias

Michael Gibson

Elisa Giovannetti

Nicolas Girard

Raffaele Giusti

P.D. Gobardhan

Tuncay Goksel

Ugur Gonlugur

Michel Gonzalez

Bruno Gori

Akiteru Goto

Mónica de Jesus Marques Grafino

Jundong Gu

Lei Gu

Jutta Guenter

Yasuo Hamamoto

Baohui Han

Joungho Han

Takashi Hanada

Akira Haro

Takaaki Hasegawa

Yoshinobu Hata

Shinya Hayashi

Yuichiro Hayashi

Zi‐Qing Hei

Rui Henrique

Rodney J. Hicks

Masahiko Higashiyama

Mitsunori Higuchi

Carlos Hinojosa

Takao Hiraki

Akira Hirasawa

Yoshihiko Hirohashi

Chung‐Ping Hsu

Po‐Kuei Hsu

Chengping Hu

Mu Hu

Chun‐Chao Huang

Jian‐An Huang

Junhui Huang

Meijuan Huang

Yu‐guang Huang

Yuan‐Chun Huang

Wei‐Heng Hung

E. Shelley Hwang

Satoshi Igawa

Shuntaro Ikawa

B.A. in ’t Hout

Kwang Ho In

Ali Inal

Minehiko Inomata

Hidetoshi Inoue

Kenichi Inoue

Nobuya Ishibashi

Genichiro Ishii

Tamotsu Ishizuka

Nabil Ismaili

Masaoki Ito

Hirotaka Iwase

Y Izumi

Matthieu Jabaudon

Andreas H. Jacobs

Dariusz Janczak

Tae‐Won Jang

Luis Jara‐Palomares

Yu Jiang

Zhengyu Jin

Zhe Jing

Rain Jõgi

David R. Jones

Philippe Joubert

Yong‐Sung Juhnn

Ki Tae Jung

Jae Jun Jung

Won Sik Jung

Vladimir Jurisic

Ikuo Kamiyama

Ozkan Kanat

Y. Altemur Karamustafaoglu

Ozan Karatag

Kaoru Kaseda

Kikuya Kato

Stamatis Katsenos

Tomoya Kawaguchi

Masaaki Kawahara

Hideki Kawai

Jun Kawai

Daigo Kawano

Larry Kedes

Umut Kefeli

Timea Keller

Kay‐Leong Khoo

Dohun Kim

Dong Soon Kim

Hee Joung Kim

HyeJin Kim

Jae Jun Kim

Junghyun Kim

Sang Hun Kim

So Ri Kim

Soo Han Kim

Sunghwan Kim

Tae Min Kim

Young‐Chul Kim

Tomoki Kimura

Masahiro Kitada

Carolyn M. Klinge

Hirosuke Kobayashi

Celalettin Kocaturk

Hyeon Kang Koh

Mikihiro Kohno

T. Kohno

Tomonobu Koizumi

Parvaiz A Koul

Aneta Kowal

Emilia Krol

Thorsten Krueger

Toshio Kubo

Kaoru Kubota

Yujin Kudo

Hideo Kunitoh

Yong‐Soo Kwon

Ava Kwong

Jung‐Nien Lai

Yeur‐Hur Lai

David C.L. Lam

Simon Law

Ilias Lazarou

Choon‐Taek Lee

Choong Sik Lee

Dae Ho Lee

Ho Yun Lee

Hyun‐Kyung Lee

Hyun‐Sung Lee

Jae Cheo Lee

Jeong Eun Lee

Jin Hwa Lee

Nam Kwon Lee

Peter N. Lee

Shih‐Chun Lee

Sung Yong Lee

Carlos Lema Rodriguez

Baolan Li

Shengqing Li

Xiaoguang Li

Xiaoyan Li

Yang Li

Evi S. Lianidou

Wen‐Chiung Lin

Yongbin Lin

Zheng‐Yu Lin

Chia‐Chuan Liu

Qiang Liu

Rong Liu

Zhe Liu

Marcela Lizano Soberon

Sihame Lkhoyaali

Simon S. Lo

Michael I. Lock

Filippo Lococo

Eudaldo Lopez‐Tomassetti Fernandez

Sonya S. Lowe

Kun Ping Lu

Marco Lucchi

Yang Luo

Yiqiao Luo

Binyun Ma

Ai Maeda

Yorinobu Maeda

Renuka Malik

Teng Mao

Paolo Marchetti

Stefano Margaritora

Athina Markou

Ronen Marmorstein

T. Matakova

Vit Matejka

Koji Matsumoto

Yukinori Matsuo

Klazien Matter

Peter Mazzone

P. J. McCall

Andrew Medford

Heta Merikallio

Rafael Meza

Koshi Mimori

Antonio Mirijello

Tempei Miyaji

Keiko Mizuno

Hiroshi Mizuuchi

Katarzyna Modrzewska

Akira Mogi

Nisha Mohindra

Yasumitsu Moriya

Giannis Mountzios

Ju‐Wei Mu

Im Il Na

Younghwa Na

Takahiro Nakajima

Yoichi Nakamura

Seiji Niho

Yuzuru Niibe

Hassan Niknejad

Cristian Oancea

Carme Obiols

Nobuaki Ochi

In‐Jae Oh

Masahiro Ohara

Takashi Ohtsuka

Hiroshi Okabe

Hironobu Okada

Tatsuro Okamoto

Mami Omori

Han Kiat Ong

Gaoliang Ouyang

Cemal Ozcelik

Mesut Özgükçe

Marianne Paesmans

Dong Il Park

Hye Lim Park

Ki‐Sung Park

Sung‐Yong Park

Manish Patel

Luis Paz‐Ares

Yang Peng

Francesco Perri

Laurence Pierrache

Jean‐Pierre Pignon

Eugenio Pompeo

Charles Powell

Qiang Pu

Bouchentouf Rachid

Mark Ragusa

Suresh Rana

Cristian Rapicetta

Ayman Rasmy

Edward A. Ratovitski

Neal E. Ready

Tommaso Ricchetti

Eduardo Rivo

Francisco Robert

Gaetano Rocco

Marco Rossato

Eunjung Ryu

Jeong Seon Ryu

Seppo Saarelainen

Tsuyoshi Saito

Ozgur Samancilar

Matteo Santoni

José Sanz‐Santos

Jiichiro Sasaki

Atsushi Sato

Yusuke Sato

Taroh Satoh

Sonja Schelhaas

A. Schmittel

Christian Schumann

Ikuo Sekine

Jae Hong Seo

Ekrem Cengiz Seyhan

Fei Shan

Kunwei Shen

Jin‐Yuan Shih

Naoto Shikama

Kimihiro Shimizu

Dong‐Yeop Shin

T Shiraishi

Hai Song

Young Jin Song

Carolina A. Souza

Elżbieta Speina

Volker Steger

David D. Stenehjem

A. Stewart

Zengliu Su

Kenichi Suda

Naoko Sueoka‐Aragane

Gee Young Suh

Ahmed Abbas Suleiman

Kenji Suzuki

Smrita Swamy

Justyna Szumilo

Federico Tacconi

Kouhei Tajima

Kazuaki Takabe

Akinori Takada

Yusuke Takahashi

Koichi Takayama

Koji Takeda

Sadanori Takeo

Nagio Takigawa

Lijie Tan

Nidhi Tandon

Chuanhao Tang

Faruk Tas

Ali Tatlı

Jin Teshima

J Ting

Trygve O. Tollefsbol

Naoto Tomita

Daniel Tong

J Turner

Hibiki Udagawa

Furkan Ufuk

Sukran Ulger

Sang‐Won Um

Ozan Usluer

Mükremin Uysal

Julio Valdivia Silva

Katrien Van Kolen

B. T. Varghese

Federico Venuta

Pierre Vera

Alain Vergnenegre

N.C. Verheuvel

Dewi Vernerey

Liza Villaruz

Andrea Viti

T Walter

Yong Wan

Jian‐Bing Wang

Jianhua Wang

Jung‐Der Wang

Junjie Wang

Mengzhao Wang

Xin Shelley Wang

Wyndham Wilson

Xin Wu

Xiujie Xie

Chong‐Rui Xu

Yan Xu

Kazuhiko Yamada

Hiromichi Yamane

Satoshi Yamasaki

Hiroki Yamaue

Huizhen Yang

Kehu Yang

Se Hoon Yang

Yunfan Yang

Shuyang Yao

Alpaslan Yavuz

Xin Ye

Douglas Yee

Seong‐Hoon Yoon

Akinobu Yoshimura

Xue Yu

Francesco Zappa

Hongmei Zeng

Zhu Zeng

Fujun Zhang

Shucai Zhang

Ya Zhang

Yushan Zhang

Wei Zhong

Wen‐zhao Zhong

Xin Zhou

Honghong Zhu

Jian‐quan Zhu

Jiang Zhu

Yimin Zhu

Wang Ziping

Xiao Zou

